# A Rare Case of Third Generation Cephalosporin-Resistant Klebsiella Meningitis

**DOI:** 10.7759/cureus.24697

**Published:** 2022-05-03

**Authors:** Adiba Zeeshan, Abdul S Ansari, Shafique Rehman, Muhammad Anzar Ullah, Aisha M Husseni

**Affiliations:** 1 Medicine, Jinnah Postgraduate Medical Centre, Karachi, PAK; 2 Medicine/Rheumatology, Jinnah Postgraduate Medical Centre, Karachi, PAK

**Keywords:** cephalosporin, klebsiella meningitis, pakistan, antibiotics, cns infection

## Abstract

Acute bacterial meningitis is one of the very common and severe forms of central nervous system (CNS) infection worldwide in almost all age groups. It remains a common cause of mortality, especially in underdeveloped countries, if not treated timely. Detecting an organism from the CSF culture is crucial in the management of acute bacterial meningitis. Selection of antibiotics according to the culture are a very important part of the management. Most commonly involved organisms include *Streptococcus pneumoniae*, *Neisseria meningitides,* and *Listeria monocytogenes*, while other organisms are very uncommon. Here, we report a rare case of *Klebsiella pneumoniae* meningitis in a young female, which was found resistant to multiple antibiotics including third generation of cephalosporin.

## Introduction

Acute bacterial meningitis is a recurrent cause of mortality in all age groups, especially in underdeveloped countries [1,2]. Erum et al. reported the highest incidence in neonates than other age groups in Pakistan [1]. It is reported as one of the top 10 causes of infection-related deaths worldwide [1]. It is characterized by fever, headache, altered level of consciousness, and meningeal irritation due to inflammation of meningeal tissues. Commonly involved organisms include *Streptococcus pneumoniae*, *Neisseria meningitides*, and *Listeria monocytogenes* [3]. However, here we report a case of *Klebsiella pneumoniae *meningitis in a 35-year-old female, an organism rarely associated with bacterial meningitis and resistant to multiple drugs.

## Case presentation

A 35-year-old female with no known co-morbidities was admitted through emergency room in the medical ward at Jinnah Postgraduate Medical Centre, a tertiary care hospital in Karachi, Pakistan. She was presented with complaints of continuous high-grade fever associated with rigors and chills, altered level of consciousness, and right-sided focal seizures for the past two days. On presentation, her blood pressure (BP) was 90/60 mm Hg, pulse was 84 beats/min, and respiratory rate was 17 breaths/min. Her Glasgow Coma Scale (GCS) was 3/15 with positive Kernig's sign and extensor plantar response. There were no focal neurological deficits. Chest, cardiovascular (CVS), and abdominal examinations were unremarkable. She was admitted with the differential diagnosis of meningitis, viral encephalitis, young stroke, or connective tissue disorders. 

Laboratory findings are summarized in Table 1. Urinalysis, blood culture, chest x-ray, and computed tomography (CT) scan of brain revealed no abnormalities. However, the cerebrospinal fluid (CSF) culture showed a few colonies of *Klebsiella pneumoniae*, which were sensitive to amikacin, gentamicin, and meropenem and resistant to other antibiotics including ampicillin, aztreonam, and third and fourth generation cephalosporin, which was another infrequent finding in this case.

**Table 1:** Laboratory findings of the patient WBC: white blood cell

Lab	Value	Reference range
Hemoglobin	10.7 g/dl	12.0-16.0 g/dl
WBC count	25.17 × 10^9^/l	5.0-10.0 × 10^9^/l
Neutrophils	95.0%	50.0-75.0%
Lymphocytes	4.0%	25.0-40.0%
Urea	38 mg/dl	10-50 mg/dl
Creatinine	1.28 mg/dl	0.5-1.5 mg/dl

Empiric treatment for suspected viral encephalitis was initiated, while the CSF results were awaited. It included IV acyclovir 500 mg Q8 hours, ceftriaxone 2 g Q12 hours, and vancomycin 1 g Q12 hours along with levetiracetam 500 mg Q12 hours for seizures. After the CSF culture and sensitivity, the report revealed the above-mentioned results, ceftriaxone and vancomycin were stopped and she was started on IV amikacin 750 mg Q12 hours, meropenem 500 mg Q8 hours, and dexamethasone 4 mg Q6 hours. Her condition improved significantly after starting this treatment and she was discharged from the hospital after full recovery on the 26th day of hospitalization.

## Discussion

Meningitis is associated with very fatal outcomes; therefore, it requires urgent medical intervention for favorable outcomes. Even with the advancements in medicine, it is still a challenge to treat this condition due to the acquired resistance to various broad-spectrum antibiotics and varying clinical presentations [2].

Of the common organisms associated with acute bacterial meningitis, Klebsiella is a rare cause that is reported in Southeast Asia but is still among the least common pathogens. In a total of 1108 patients identified with meningitis in a study by van de Beek et al., *Streptococcus pneumoniae* and *Neisseria meningitis* were the most commonly found organisms [4]. Moreover, in another study, Klebsiella was the least commonly isolated organism in confirmed cases of bacterial meningitis [2]. And, only two cases of community-acquired Klebsiella meningitis have been reported in the Caribbean [5]. 

Studies have associated Klebsiella meningitis with preceding diseases like diabetes, disseminated intravascular coagulation, and liver abscesses [3,6]. However, despite a comprehensive medical, surgical, and family history along with the examination and a series of tests, this patient had neither of these conditions or any other associated co-morbidities. The source of this rare organism in a completely healthy patient with no past medical history is still unknown. A study has revealed ear infections like otitis media as a possible cause of spread of Klebsiella to the CNS causing meningitis, but the patient did not report any such symptom nor could recall any ear infection in the past [7]. The initial treatment prescribed was analogous to the typically prescribed treatment for a suspected case of a CNS infection [8].

It is advisable to continue the antibiotics for a period of 14-21 days depending on the response of the patient [3]. In our case, the treatment continued for 14 days. Fortunately, this patient had a very positive clinical outcome. She was discharged completely healthy after a 26-day stay at the hospital. The reason for this outcome was prompt and aggressive medical intervention right after admission. Initiating prompt treatment in correlation with the Glasgow Coma Scale is very important in this disease; prescribed antibiotics, age, gender, disease severity, and even antibiotic-resistant isolates seemed to have no role in determining the outcome [9]. It was only the time of administration of the first antibiotic dose that was a significant predictor and this is where we were at an advantage. It is advisable to repeat the CSF analysis after getting cured in cases of multidrug-resistant organisms like this case [3]. But unfortunately, the patient refused a follow-up due to personal reasons.

## Conclusions

This patient had a very positive clinical outcome which was due to prompt and aggressive medical intervention right after admission. Therefore, it is very important to timely diagnose and treat life-threatening conditions like meningitis, especially when it involves rare organisms that are multidrug-resistant.
